# Comparing gene set analysis methods on single-nucleotide polymorphism data from Genetic Analysis Workshop 16

**DOI:** 10.1186/1753-6561-3-s7-s96

**Published:** 2009-12-15

**Authors:** Nathan L Tintle, Bryce Borchers, Marshall Brown, Airat Bekmetjev

**Affiliations:** 1 Department of Mathematics, Hope College, 27 Graves Place, Holland, Michigan 49423, USA; 2 Department of Mathematics, Rose-Hulman Institute of Technology, 5500 Wabash Avenue, Terre Haute, Indiana 47803, USA; 3 Department of Mathematics, Seattle Pacific University, 3307 Third Avenue West, Seattle, Washington 98119, USA

## Abstract

Recently, gene set analysis (GSA) has been extended from use on gene expression data to use on single-nucleotide polymorphism (SNP) data in genome-wide association studies. When GSA has been demonstrated on SNP data, two popular statistics from gene expression data analysis (gene set enrichment analysis [GSEA] and Fisher's exact test [FET]) have been used. However, GSEA and FET have shown a lack of power and robustness in the analysis of gene expression data. The purpose of this work is to investigate whether the same issues are also true for the analysis of SNP data. Ultimately, we conclude that GSEA and FET are not optimal for the analysis of SNP data when compared with the SUMSTAT method. In analysis of real SNP data from the Framingham Heart Study, we find that SUMSTAT finds many more gene sets to be significant when compared with other methods. In an analysis of simulated data, SUMSTAT demonstrates high power and better control of the type I error rate. GSA is a promising approach to the analysis of SNP data in GWAS and use of the SUMSTAT statistic instead of GSEA or FET may increase power and robustness.

## Background

Gene set analysis (GSA) methods of analyzing genome-wide gene expression data (DNA microarray) are increasingly popular [1-12]. GSA techniques view the statistical significance of sets of genes, instead of examining significance on a gene-by-gene basis. GSA is purported to have more power to detect subtle, but consistent, changes in expression levels than gene-by-gene significance tests of gene expression data.

Recently, Wang et al. [13] and Chasman [14] have proposed applying GSA methods to SNP data in genome-wide association studies, providing a new method to address one of the biggest challenges facing genome-wide single-nucleotide polymorphism (SNP) studies today: namely, a lack of sufficient power to detect small effects as significant [15]. Instead of focusing only on the SNPs that are most significant as would be done in a standard analysis, GSA evaluates sets of SNPs for significance by first associating SNPs with genes. Genes are then grouped into biologically meaningful sets (e.g., genes in the same cytogenetic band or pathway). Typically, many of the gene sets tested overlap (i.e., genes, and thus SNPs, are in multiple gene sets). The statistical significance of each set of SNPs/genes is then computed. In their implementation of GSA, Wang et al. [13] chose what is arguably the most popular of the GSA statistics: the weighted Kolmogorov-Smirnov-like running-sum statistic of gene set enrichment analysis (GSEA) [2,3]. However, increasing evidence in the analysis of gene expression data suggests that the GSEA statistic may not be optimal compared with other methods. Efron and Tibshirani [5], Dinu et al. [4], and Tintle et al. [12] have all argued that GSEA has less power than other test statistics. To address the shortcomings of GSEA, Efron and Tibshirani proposed the MAXMEAN statistic [5] and Dinu et al. proposed SAM-GS [4]. Tintle et al. [12] compared these methods and found that the MAXMEAN statistic provides increased power compared with SAM-GS and GSEA. Chasman [14] followed similar methods as Wang et al. [13] while also comparing GSEA to the hyper-geometric distribution/Fisher's exact test (FET) method. Chasman [14] found that FET was more powerful for sets containing a few highly significant genes, while GSEA was more powerful for sets containing many more weakly associated genes. However, FET has been criticized by others due to a lack of robustness and low power when compared with other statistics [9,12].

Because GSEA and FET have been demonstrated to be less than optimal for gene expression data, in the following analysis we compare alternative GSA statistics (based on MAXMEAN and SAM-GS) following the general outline of the Wang et al. method [13] using SNP and phenotype (real and simulated) data available from the Framingham Heart Study as part of Genetic Analysis Workshop 16 (GAW16).

## Methods

### Obtaining gene sets

In general, we followed the methods of Wang et al. [13] to assign SNPs to gene sets. Approximately 550,000 SNPs were available for analysis. SNPs were screened to ensure a minor allele frequency >5%, consistency with Hardy-Weinberg equilibrium (*p*-value for goodness of fit test > 0.001), and less than 10% no calls. The remaining SNPs were tested for association with each of the two phenotypes of interest (diabetes and heart disease) using a standard χ^2 ^test of association. We then used the Ensembl database [16] to create a list of all known human genes. Each SNP was assigned to the gene closest to it, as long as the closest gene was within 500 kb of the SNP. Each gene was then assigned a statistic equal to the largest χ^2 ^statistic of the SNPs associated with that gene. Gene sets (assignments of genes to biologically meaningful groups) were then downloaded from the Broad Institute's MsigDB [3]. Gene sets considered here are a portion of all those available from MsigDB. Specifically, we consider 306 positional (cytogenetic band) gene sets and all 396 gene sets based on the Gene Ontology's "molecular function" classification.

### Statistical analysis

In order to evaluate the statistical significance of sets of genes, we compared the GSEA statistic used by Wang et al. [13] to three other statistics considered in the literature. To aid in the description of the different statistics, let *t*_1_, *t*_2_, ..., *t*_*r *_represent the χ^2 ^test statistics for each of the *r *genes in the gene set. In order to compute the GSEA-like test statistic we follow the method of Subramanian et al. [3] and Wang et al. [13]. In essence, the statistic is a weighted Kolmogorov-Smirnov-like running sum statistic, where the "weight" is *t*_*i *_for the *i*^th ^gene. Two of the other gene set test statistics considered were SUMSTAT() (based on MAXMEAN [5]) and SUMSQ () (based on SAM-GS [4]). Lastly, the FET method was considered [14]. FET first classifies each gene as either "significantly associated with the phenotype" or not, and then compares the proportion of significant genes in the set of interest with the proportion of significant genes not in the set of interest using Fisher's exact test. In order to decide if a gene was significantly associated with the phenotype, we used χ^2 ^(1 d.f.) quantiles as cutoffs (5.992, 9.210, 13.816, and 18.421) for the individual gene test statistics. Each statistic (GSEA, SUMSTAT, SUMSQ, and FET) was then computed on each gene set for each of the two phenotypes of interest. For GSEA, SUMSTAT, and SUMSQ, the observed statistics were compared with the same statistics computed on 1000 randomly selected gene sets containing the same number of genes as the set of interest. The *p*-value of each set was then computed as the fraction of times the observed statistic was greater than the statistic based on the random sets. Finally, a false-discovery rate (FDR) procedure (5%) was used to adjust for multiple testing of multiple gene sets.

### Sample

Data on participants in the Framingham Heart Study was analyzed using data from GAW16 Problems 2 and 3. To simplify analyses, the sample provided was reduced to unrelated individuals as described below. There were 6525 individuals for whom there was genotype and phenotype information and who were also in pedigrees. We selected a single person to represent each pedigree. To increase the number of cases in the sample, we selected individuals to represent a family if they had heart disease, diabetes, or were the oldest in the family (in that order of preference), leaving 730 individuals. In addition to the 6525 individuals in pedigrees, there were 227 genotyped and phenotyped individuals who were singletons. We combined all genetically unrelated individuals (730+227), leaving a total analysis sample of 957. Of the 957 individuals, 158 have ever had a heart disease diagnosis and 167 have ever had diabetes.

## Results

### Comparing different test statistics on Framingham Heart Study data

**Figure 1:**
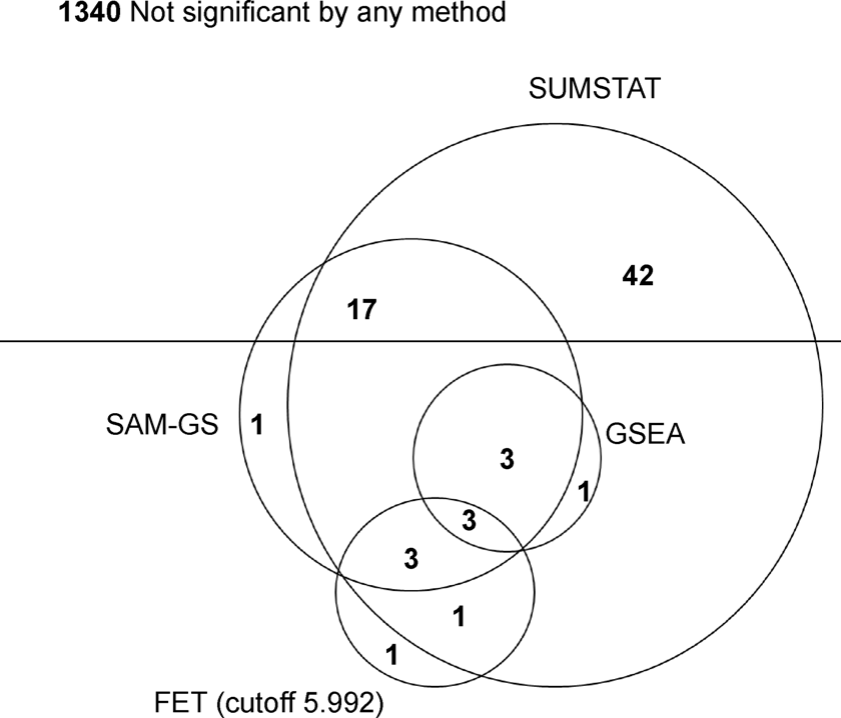
**Venn diagram of sets identified as significant by four different GSA methods for Framingham Heart Study data**. The numbers represent the significant sets in each non-overlapping region. Total number of sets depicted is 1412, which is 706 gene sets for each of the two phenotypes. There were 1340 sets not identified as significant by any method.

After computing the *p*-value of each of the 706 gene sets for each of the two phenotypes using the different statistical methods (GSEA, SUMSTAT, SUMSQ, and FET), a FDR of 5% was applied to determine significance. Overall, the SUMSTAT method identified 70 sets as significant, SUMSQ identified 27, GSEA identified 7, FET with a 5.992 cutoff identified 8, and the FET with three other cutoffs identified 0 sets as significant. As represented in Figure 1, 26 of the 27 significant sets identified by SUMSQ, 7 of the 8 FET (5.992 cutoff), and all 7 of the sets identified by GSEA as significant were also identified as significant by the SUMSTAT method.

### Comparing different test statistics on simulated data

Because simulated phenotype data were not available for all individuals in the real data sample, in the simulated data analysis there are 876 individuals. Of these, there are 210 who have ever had a (simulated) myocardial infarction and 666 who have not. Two types of gene sets were considered in the analysis of the simulated data. First, the cytogenetic band sets and the GO-Molecular Function sets were used. Each set was identified as containing no, some weak (1-9), many weak (10+) genes, or one to two strongly associated gene. Second, pseudo-gene sets were created to contain large numbers of genes associated with the phenotype. Both the real and pseudo-gene sets were then analyzed using the same procedures as for the real data analysis. In the simulated data analysis 2000 SNPs were weakly associated with myocardial infarction or a related phenotype (e.g., high-density lipoprotein cholesterol level), while 19 were more strongly associated.

Table 1 shows that gene sets containing weakly associated genes were more likely to be identified as significant (*p *< 0.05; 1000 permutations) by the SUMSTAT method than by SUMSQ, GSEA, or FET with any cutoff, regardless of whether the gene sets were real or pseudo-gene sets. Pseduo-gene sets containing strongly associated genes were best identified by SUMSTAT, SUMSQ, or FET with a cutoff of 18.421. None of the methods performed well at finding major genes when there was only one or two of them in a set. We note that the optimal FET cutoff reverses when comparing pseudo-gene sets containing weak or strongly associated genes. All methods controlled type I error rates for both pseudo-genes and real sets of genes.

**Table 1:** Percent of sets found as significant for the simulated data

	FET						
				
	5.9	9.2	13.8	18.4	GSEA	SUMSQ	SUMSTAT
Pseudo-gene sets							
No associated genes	1.4	2.8	0.0	0.9	3.2	3.7	3.7
1-9 weakly associated genes	9.8	3.3	1.6	3.3	8.5	8.2	8.2
10+ weakly associated genes	10.6	7.7	1.9	1.9	11.5	11.5	15.4
1-2 strongly associated genes, but no weakly associated genes	0.0	0.0	5.3	0.0	0.0	0.0	0.0
Real gene sets							
Many weakly associated genes	51.7	49.2	13.3	5.0	58.3	60.0	70.8
Some strongly associated genes	2.5	3.8	7.5	33.8	6.3	36.3	23.8
Null sets (no associated genes)	0.0	6.7	1.7	3.3	1.7	5.0	3.3

### Results of gene set analysis on Framingham data

Based on Figure 1, Table 1, and previous findings by Efron and Tibshirani [5] and Tintle et al. [12], the SUMSTAT method appears to provide the most powerful and robust results, so we only provide a detailed set of SUMSTAT findings here (Tables 2 and 3).

**Table 2:** The cytogenetic band sets found to be significant by SUMSTAT (FDR 5%)

**Diabetes**
2q34
2q36
3p14
3p26
4q22
4q32
5q14
5q23
5p14
9p24
9q21
10p14
10p15
11q21
12p12
12q23
13q12
13q22
14q13
18q12
18q21
18q22
**Heart Disease**
1p31
2q24
3p26
4p15
5p13
6p24
6p25
9p24
9q
9q21
10p12
10p15
12q15
18q21
18q22
20p12
21q21

**Table 3:** The molecular function gene sets found to be significant by SUMSTAT (FDR 5%)

**Heart Disease**
Cation Transmembrane Transporter Activity
Glutamate Receptor Activity
Hematopoietin Interferon Class D200 Domain Cytokine Receptor Activity
Ionotropic Glutamate Receptor Activity
Low density lipoprotein activity
Sialyltransferase Activity
Transmembrane receptor protein kinase activity
**Diabetes**
Cyclic nucleotide phosphodiesterase activity
G-protein coupled receptor activity
Gated Channel activity
Glutamate receptor activity
GTPase regulator activity
Guanyl nucleotide exchange factor activity
Ionotropic glutamate receptor activity
Lipoprotein binding
Low-density lipoprotein binding
Phosphoric diester hydrolase activity
Phosphoric ester hydrolase activity
Transmembrane receptor protein phosphate activity
3-5-cyclic nucleotide phosphodiesterase activity
Cation channel activity
Interleukin binding
GTPase activator activity
Ion transmembrane transport activity
Phosphoprotein phosphatase activity
Gaba receptor activity
Metal ion transmembrane transporter activity
Protein tyrosine phosphatase activity
Growth factor binding
Metabotropic glutamate gaba-b like receptor activity
Delayed rectifier potassium channel activity

## Conclusion

GSA offers a promising approach to genome-wide studies. Recently, Wang et al. [13] and Chasman [14] extended the GSA methodology from DNA microarrays (gene expression data analysis) to genome-wide SNP data. However, recent evidence suggests that the statistics selected by Wang et al. (GSEA) [13] and Chasman [14] (FET/GSEA) are less powerful and robust then other methods when analyzing gene expression data. In this paper we have presented evidence that this limitation also holds true for analysis of real and simulated SNP data. The SUMSTAT method found many more sets to be significant than the other methods while controlling the type I error rate. The FET method was also shown to lack robustness to different types of sets (strong or weakly associated genes), an inherent limitation of an approach that requires choosing an arbitrary cutoff.

As pointed out by Wang et al. [13], the method used here to assess significance (random gene sets) is inherently biased due to assignment of the maximum SNP statistic to the gene. However, the analysis here, the results of Wang et al. [13], as well as results in other papers [12], all find that assessing significance with random gene sets provides reasonable results. In addition to the random set approach, Wang et al. [13] use a more traditional subject permutation strategy to assess significance. Goeman and Buhlman [10] as well as Efron and Tibshirani [5] provide clear and helpful discussions of the implications of the different strategies for assessing significance in GSA.

A potential concern in GSA is the linkage disequilibrium structure of the genes in the set. GSA, as implemented for genome-wide association studies, ignores gene-gene correlation. However, as argued by Wang et al. [13], this is only an issue if the genes overlap the same linkage disequilibrium block or have an epistatic interaction. In these cases GSA will overestimate significance of gene sets.

Lastly, in the analysis presented here, sample sizes are relatively small. Larger sample sizes would increase the precision of initial SNP association tests and, thus, increase the power of the related tests. In addition to sample size, the power of tests in GSA is related to the number of genes in the gene set and the size of the shift in distribution of statistics between the genes in the set compared with those not in the set. Further work is necessary to fully explore potential modifications to the current methods of GSA in order to maximize their power in analyzing genome-wide association data.

The analysis presented here provides additional evidence that the use of GSEA for pathway-based testing in SNP genome-wide association studies is less than optimal. Using the SUMSTAT statistic in lieu of the GSEA statistic offers a promising step forward in GSA of genome-wide SNP data.

## List of abbreviations used

GAW16: Genetic Analysis Workshop 16; GSA: Gene set analysis; GSEA: Gene set enrichment analysis; FDR: False-discovery rate; FET: Fisher's exact test; SNP: Single-nucleotide polymorphism.

## Competing interests

The authors declare that they have no competing interests.

## Authors' contributions

NLT and AB helped conceive of the study and mentored BB and MB in carrying it out. BB and MB implemented and conducted all preliminary analyses. Final analyses and drafting the manuscript was done by NLT. All authors read and approved the final manuscript.
